# Survey on Knowledge of First Aid Management of Burns Amongst Medical and Non-medical Students in Karachi, Pakistan: Need for an Educational Intervention?

**DOI:** 10.7759/cureus.6674

**Published:** 2020-01-16

**Authors:** Ramsha Riaz, Lubna Riaz, Jehanzeb Khan, Mariam Baloch

**Affiliations:** 1 Internal Medicine, Dow Medical College, Dow University of Health Sciences (DUHS), Karachi, PAK; 2 Forensic Medicine & Toxicology, Dow Medical College, Dow University of Health Sciences (DUHS), Karachi, PAK; 3 Internal Medicine, Civil Hospital Karachi, Dow University of Health Sciences, Karachi, PAK; 4 Medicine, Dow Medical College, Dow University of Health Sciences, Karachi, PAK

**Keywords:** burns, burns management, burn injuries, burns first aid, burn first aid treatment, undergraduate, knowledge, pakistan

## Abstract

Background: Burn related injuries (BRIs) are relatively common, mostly accidental, and highly preventable forms of injury. First aid management of BRIs can have a significant impact on the outcome and morbidity of these injuries, yet there seems to be an inadequacy in the knowledge related to burn management worldwide. Hence, our study aimed to delineate the level of knowledge and awareness of burn first aid treatment (BFAT) amongst undergraduate students, and the impact training courses had on that knowledge.

Methods: A cross-sectional study was conducted by employing undergraduate medical and non-medical students from various universities of Karachi, Pakistan. By means of convenience sampling, 400 students were sent online, structured questionnaires. The analysis was conducted using Statistical Package for Social Sciences (SPSS version 23.0, IBM Corp., Armonk, NY, US), and associations calculated through t-tests. A mean knowledge score was calculated to assess the participant’s adequacy of knowledge regarding BFAT.

Results: Medical students had a better knowledge score than their non-medical counterparts (5.8 ± 1.6 versus 3.6 ± 1.5, *P* < 0.01), however, overall knowledge of BFAT remained inadequate amongst participants. Students who received formal training scored a higher mean knowledge score than the majority of students with no training (*P* < 0.01).

Conclusion: The majority of students had insufficient overall knowledge about BFAT, highlighting the need for integrating this topic into the curriculum. In addition, education of the masses via multimedia and conducting formal training courses, are both imperative in raising awareness and reducing the occurrence of BRIs.

## Introduction

Burn related injuries (BRIs) are said to take place when part of or all of the layers of the skin are disrupted by hot liquids (scalds), by hot solids (contact burns), or by a flame (flame burns); moreover, injuries to the skin caused by radiation, electricity, or chemicals are also classified as burns [1]. Socio-environmental advancement and modernization have led to an increase in the number of accidents in our daily lives, with BRIs constituting the major portion of such events.

Injuries related to burns and fires account for more than 300,000 deaths annually throughout the world [2]. According to the American Burn Association (ABA), more than 450,000 individuals present to the emergency departments, clinics, or physician’s offices to receive treatment for a burn-related injury in the United States and Canada; both of which are high-income and developed countries [3]. The vast majority of these burns are not fatal, not lethal, and preventable. Also notable is the fact that even though the morbidity and mortality due to fire and flames has declined worldwide over the years, 90% of deaths caused by burns and fires still occur in low- and middle-income countries (LMIC), where burn prevention programs are not common and quality of acute management is inconsistent [2].

In Pakistan, the absence of a National Burn Registry program and the under-reported nature of BRIs, make it difficult to estimate the annual burden of injuries related to burns and fires presenting to hospitals and to the handful of specialized burn centers located in urban areas [4]. Only a few studies have aimed to determine the incidence of burn injuries in our country, with one study reporting an incidence rate of 147 per 100,000 patients presenting to the emergency departments (EDs) of seven tertiary care hospitals [4]. Moreover, the mortality rate associated with burn and fire injuries in Pakistan has ranged from as low as 6.5% to as high as 41.3% [4-5].

The majority of the most common BRIs occur within the household, which means that the first respondents in these cases are usually the family members, friends, etc [1,5-6]. Hence, to be able to manage these common, non-fatal burns, it is important to have a clear understanding regarding burn first aid treatment (BFAT), because the provision of optimal first aid treatment can significantly decrease tissue damage, fasten wound re-epithelialization, and reduce associated scarring [7]. On the other hand, inadequate knowledge and incorrect application of techniques can not only influence the outcome of the injury, but may also hinder proper recovery, and at worst, exacerbate the damage caused by the initial injury.

Several studies have been conducted worldwide to demonstrate the adequacy of knowledge regarding BFAT, spanning across a variety of ages and groups. A majority of these studies have shown a lack of understanding and knowledge of BFAT in groups such as medical students and healthcare workers in the United Kingdom; the Saudi population; students in Nigeria; and adults in Australia [8-12]. A limited number of studies on this topic; however, have been carried out in Pakistan, where one study revealed an overall lack of knowledge of BFAT amongst the general population of Rawalpindi, Pakistan, while another study demonstrated inadequate knowledge in handling burns amongst parents of children suffering from burns [13-14]. Data has also further shown that factors such as the educational status of individuals, previous knowledge of first aid of burns, and having attended a first aid course/training are associated with higher levels of knowledge scores in the respective studies [11-15]. This reflects that imparting the knowledge and basic training regarding BFAT should be a point of focus to not only students belonging to schools, colleges, and universities, but also to the general population.

Keeping this in mind, the aim of our study was to delineate the level of knowledge and awareness of burn first aid management amongst medical and non-medical students and to highlight the urgent need of addressing gaps, if any, in this knowledge. We also aim to assess the impact of attending first aid training and/or courses on the knowledge of BFAT, and hence, demonstrate the usefulness of implementing them as part of the student’s curricula.

## Materials and methods

This descriptive, cross-sectional study was conducted over a time duration of four months, from July 2019 to October 2019; employing students from various medical and non-medical universities of Karachi, Pakistan. The sample size was obtained via OpenEpi.com sample size calculator. Using an anticipated frequency (*p*) of 83%, at a confidence interval (CI) of 95%, a minimum sample size of 217 was calculated [13].

To assess the knowledge and awareness of burn-related injuries and their first aid management, a structured, self-reported questionnaire was drafted after thorough research of the literature available on this topic [1,7,9-10,16]. The questionnaire included general demographic details of the participants such as age, gender, and education. The subsequent section included 10 questions evaluating their knowledge on first aid, including knowledge of the sources of burns, most common location of burn occurrence, most important contributor to the severity of burns, best initial step taken immediately after a burn, optimum time for cooling of the burnt area, action taken in case a person is wearing jewelry/ornaments, action taken when a blister forms on the burnt area, action taken when a person incurs burns on the face/neck region, pain management of minor burns, and the action taken when a person is on fire. The participant’s confidence levels in administering BFAT were also assessed using a Likert five-point scale.

Furthermore, the questionnaire also inquired whether the participants had received any formal training or attended any educational workshop on the management of burns, and regarding the sources contributing to their existing knowledge of burns (such as their educational institute, books, journals, radio, television, Internet, etc). For the purpose of our research, formal training referred to any workshop, seminar, simulation, or first aid course directed toward BRI and their appropriate management.

Informed consent was obtained from all participants, and their confidentiality and anonymity were maintained. Initially, a pilot study was conducted by employing 20 participants and making use of their suggestions to enhance the clarity of the final questionnaire. The questionnaire was then made online through Google forms, and sent to up to 400 medical and non-medical students belonging to various universities of Karachi, by means of convenience sampling. Incompletely filled and unacceptable forms were discarded and we were able to obtain a total of 346 complete forms. All the data obtained were then transferred onto Microsoft Excel 2016, and statistical analysis was conducted through Statistical Package for Social Sciences (SPSS version 23.0, IBM Corp., Armonk, NY, US).

The participants were scored on a 10-point scale (one point for each correct answer on the burn injury-related questions), and their knowledge scores were categorized as Poor (<50%), Fair (50%-70%), and Excellent (>70%) [17]. To compare the mean knowledge scores of medical and non-medical students, an independent sample t-test was applied and a *P*-value < 0.05 was considered statistically significant. The association between formal training and mean knowledge scores was also obtained using t-test. All data were presented in the form of frequencies and percentages, and chi-square test was employed to calculate associations between categorical variables.

## Results

Our study comprised a total of 346 participants, more than half of which were medical students (56.6%). The demographics of the study participants are summarized in Table 1. 

**Table 1:** General demographics of the study participants (n = 346) SD: standard deviation ^a^Calculated using chi-square for categorical data, and independent sample t-test for continuous data; *P*-value < 0.05 considered statistically significant.

	Medical Students	Non-Medical Students	P-value^ a^
Field of education (%)	196 (56.6)	150 (43.4)	
Gender			
Males	58	93	<0.05
Females	138	58	
Mean age (years) ± SD	21.8 ± 1.2	22.0 ± 2.2	0.43

Table 2 compares the mean knowledge scores and confidence levels in administering burn first aid between medical and non-medical students. Our data revealed that there was a statistically significant difference between the two knowledge scores (*P* < 0.01), and medical students had a better knowledge score than their non-medical counterparts (5.8 ± 1.6 and 3.6 ± 1.5, respectively). None of the non-medical students out of a total of 150 scored >70%, as opposed to 30 medical students scoring above this threshold. Irrespective of the knowledge scores obtained, majority of the medical students were “Moderately Confident” (34.7%) in administering first aid, while majority of the non-medical students said they were “Confident” (30.7%). Overall, majority of the students combined were “Slightly Confident” (105/346, 30.3%) in their ability to administer first aid. Furthermore, there was a statistically significant difference in the number of students who had received formal training from both the groups (*P* < 0.01). Overall, a staggeringly high number of students had never received any form of formal training regarding BRI or their management (262/346, 76%). While 41% of medical students had still received some form of training, the percentage was even worse for non-medical students (1%). 

**Table 2:** Mean knowledge scores of medical students and non-medical students SD: standard deviation ^a^Calculated using an independent sample t-test for continuous data and chi-square for categorical data; *P*-value < 0.05 considered statistically significant.

	Medical Students	Non-Medical Students	P-value^ a^
Mean knowledge score ± SD (%)	5.8 ± 1.6	3.6 ± 1.5	<0.01
Poor (<50%)	45 (23)	106 (71)	<0.01
Fair (50%-70%)	121 (62)	44 (29)	<0.01
Excellent (>70%)	30 (15)	0 (0)	<0.01
Confidence level in administering burn first aid (%)			
Not confident at all	27 (13.7)	29 (19.3)	0.16
Slightly confident	66 (33.7)	39 (26.0)	0.12
Moderately confident	68 (34.7)	28 (18.7)	<0.01
Confident	23 (11.7)	46 (30.7)	<0.01
Very confident	12 (6.1)	8 (5.3)	0.76
Received formal training (%)			
Yes	81 (41)	2 (1)	<0.01
No	115 (59)	148 (99)	

Table 3 reveals the impact that formal training had on mean knowledge scores of students. The 24% of students who had received some form of formal training scored a higher and better mean knowledge score than the majority of the students with no training (6.4 ± 1.5 and 4.4 ± 1.8, respectively; *P* < 0.01). 

**Table 3:** Association of formal training with mean knowledge scores SD: standard deviation ^a^Calculated using independent sample t-test; *P*-value < 0.05 considered statistically significant.

	Yes	No	P-value^a^
Received formal training (%)	83 (24)	262 (76)	
Mean knowledge score ± SD	6.4 ± 1.5	4.4 ± 1.8	<0.01

**Figure 1:**
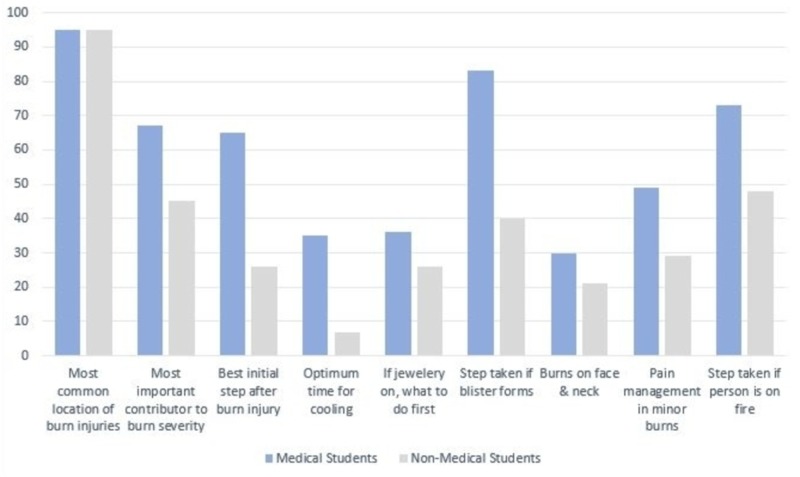
Percentage of correctly identified responses by medical students and non-medical students

Figure 1 shows the individual responses of participants to questions assessing their burns related knowledge, where medical students performed better in eight out of nine responses. 

**Figure 2:**
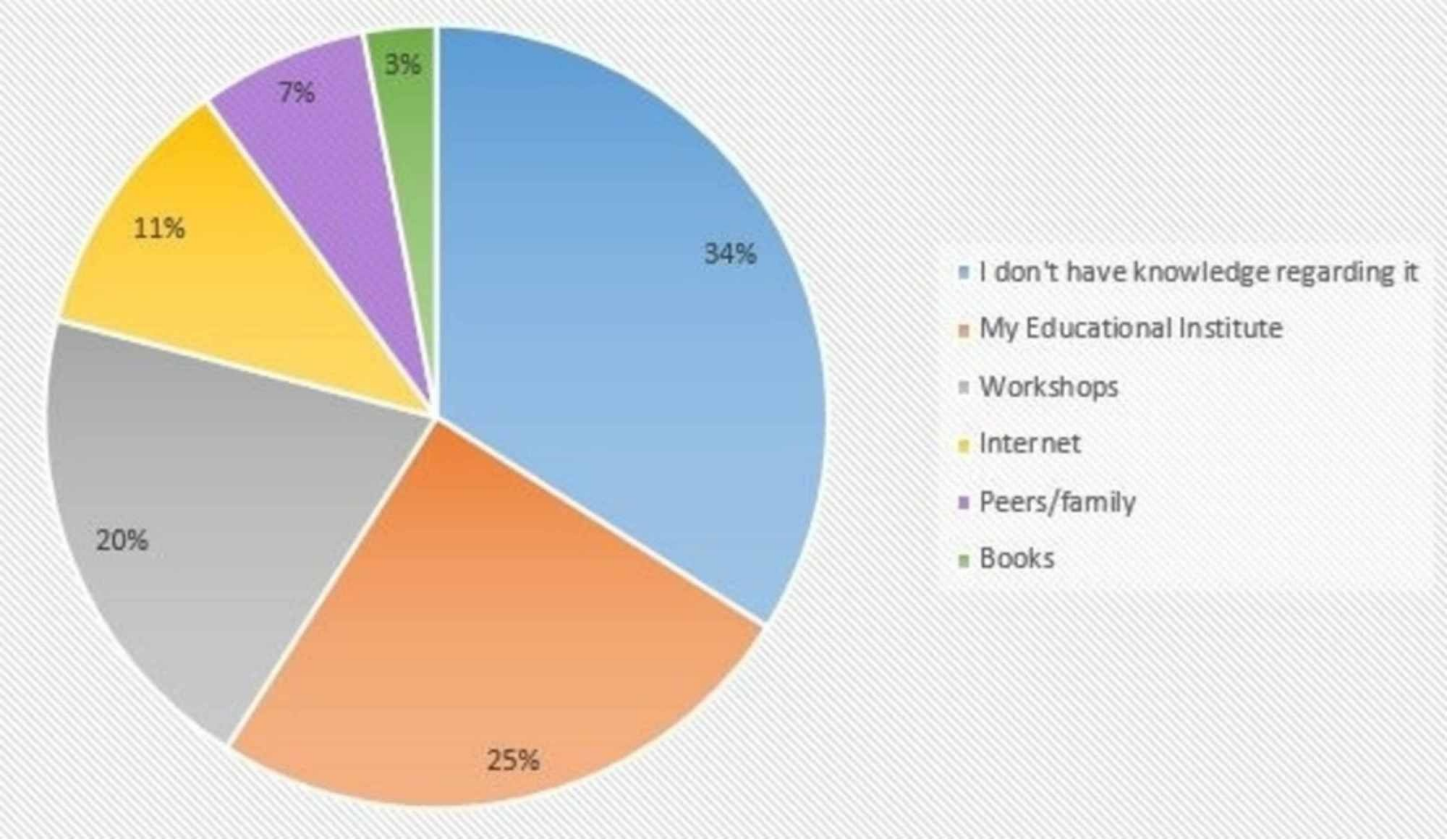
Sources of knowledge regarding first aid management of BRIs

Almost one-third of the students claimed to have no knowledge regarding first aid management of BRIs. A quarter of the study participants attributed their educational institute as their main source of knowledge, while the least contributing source was found to be books/journals (3%). These findings are demonstrated in Figure 2. 

## Discussion

BFAT is an important component of burn management and a significant determinant of the outcome and morbidity related to burn injuries. When carried out properly, it not only influences and fastens healing but also minimizes the need for surgical intervention and the added economic burden on the healthcare sector [18]. Studies conducted worldwide have shown poor knowledge of first aid for burns, which is why our study aimed to assess the knowledge of BFAT amongst the young generation of our nation, and the need of an educational intervention for the general population with respect to this topic. 

Our study demonstrated an overall lack of knowledge regarding BFAT as is evident by poor mean knowledge scores, with 44% of total students scoring poorly (<50%), and only 9% of students scoring >70% (“Excellent”). These findings are consistent with other studies carried out in both developed and less developed parts of the world [8-12]. In the survey of Wallace et al. (2013) conducted in Australia, 30%-50% of incorrect responses to various burn-related scenarios were observed, with the scores being improved by 14.5%-17.2% in participants who had undertaken a first aid course in the last five years [12]. Another study conducted in the United Kingdom reflected subpar knowledge of BFAT amongst healthcare workers [9]. The situation in Pakistan also follows this global streak, since our results were comparable with other studies conducted in Pakistan in terms of inadequate knowledge regarding BFAT [13-14].

Apart from highlighting the overall low knowledge scores, our study also delineates the difference in the performance of medical and non-medical students. Medical students were shown to have higher knowledge scores (*P* < 0.01) than their non-medical counterparts, with a majority of them obtaining Fair (50%-70%) scores. This is consistent with another study, where a majority of the medical students from Saudi Arabia obtained fair knowledge scores (between 50% and 75%) [17]. This finding may be attributed to the fact that medical students, owing to their clinical and scientific background, and probable exposure to burn-related cases during the course of their studies, managed to achieve a higher number of correct answers. Whereas, non-medical students usually have little to no exposure regarding first aid management in their daily lives or in their curricula, which may account for their poorer performance. However, it is important to note that the scores of medical students, albeit higher than non-medical students, do not stray from the general trend of healthcare workers having inadequate knowledge of first aid of burns [8-9].

An interesting aspect to note is the confidence level of performing first aid. Ironically, a greater number of non-medical students, who had lower mean knowledge scores, were observed to be more confident (30.7%) when questioned about administering burn first aid than medical students (11.7%). This figure is even lower than that of another study which reported that only 32% of medical students felt confident in managing a burn patient [8]. The statistically significant contrast clearly points out the limited grasp of non-medical students on the subject of BFAT and the consequent overestimation of their skill set. On the other hand, the low confidence of medical undergraduates concurs with prior statistics, and the unpreparedness and uncertainty may be associated with inadequate clinical exposure, specifically in burns [8].

Apart from highlighting knowledge scores, it is also important to focus on individual responses to burn-related questions, so our educational approach can be more focused. Regarding the question on the best step after incurring a burn injury, a greater number of medical students (65%) chose the correct response of "cooling under running tap water," than non-medical students (26%) [6,9,14,16]. Having the correct knowledge of the first and most basic step is crucial because using alternative methods such as ice/ice packs/ice water can result in an increased risk of tissue damage and tissue necrosis, and risk of hypothermia if used in larger quantities for a prolonged time [6,16,19]. Similarly, applying traditional home remedies like toothpaste, mud, butter, etc. can not only contaminate the wound and trap heat inside the tissue but also make the initial assessment of the severity and depth of the burn difficult [6,19].

Concerning the optimal cooling time of the burn area, only 35% of medical and a measly 7% of non-medical students answered correctly ("20 min"), which can be compared to the 10.2% correct responses in the survey by Siddiqui et al. [9-10,17]. It should be noted that incorrect responses may solely not be due to the lack of information but rather an inconsistency in the data available. For instance, the variability in the literature regarding the optimal time of cooling ranges from 10 to 30 minutes and leads to a multitude of “correct” answers [6,8]. Unsurprisingly, the response to the next step after blister formation was answered mostly correct ("leave them until you seek medical assistance") by medical students with a stark gap in a number of correct responses when compared to non-medical students [1,16]. Beliefs like puncturing them and application of toothpaste were common and need to be addressed in the future to get rid of common misconceptions, because such actions may exacerbate the initial injury. Contrary to the study of Mishra et al., where a majority of the participants (82.3%) knew the correct answer of removing jewelry and accessories from the burnt area, students in our survey responded poorly [13,16]. Less than half of medical students marked the right answer which is alarming and needs to be addressed since accessories not only get in the way of injury, but the following edema makes the future removal difficult and painful. Concerning the “stop, drop, and roll” mantra in case the clothes catch on fire, the response of medical students was found to be adequate (73%) and comparable to the results of a study based on medical students in Saudi Arabia [17].

One of the most significant findings of our study was the positive impact of burn first aid training on the mean knowledge scores of the participants. Students who received some form of formal training obtained higher mean knowledge scores (*P* < 0.01), which can be comparable to other studies revealing similar findings [11-12,14]. In the study by Wallace et al., 15% more participants provided correct responses if they had previously attended a first aid course, as compared to those who had not [12]. Likewise, in Vietnam, a marked improvement was witnessed in the emergency management skills of burn injuries amongst healthcare providers after the provision of appropriate burn-related training courses [20]. This highlights the importance of not only introducing proper first aid training programs for students but also making it a mandatory part of their curricula. Another study conducted in Nigeria, a developing country, showed that students who had prior knowledge of BFAT had relatively better knowledge scores than those who didn’t [11]. This demonstrates that even in resource-limited environments, education plays a crucial role in imparting correct knowledge about lifesaving skills.

Apart from conducting formal training programs and workshops, another effective method of reaching the masses could be via the use of multimedia to raise awareness regarding correct steps of BFAT, eradicate common misconceptions, and address harmful practices. A similar approach was followed and studied by Skinner et al. in New Zealand, where a mass multimedia campaign making use of television, radio, billboards, etc. focused on adequate burn first aid practices and eventually led to a substantial drop in hospitalization and operative procedures, and improvement in BFAT adequacy [21]. In a study by Anwer et al., it was demonstrated that most of the burn-related injuries were accidental and occurred as a result of negligence and lack of awareness [22]. Hence, taking effective measures to educate and/or train the masses can prove to be beneficial in reducing BRIs, and improving their overall outcome.

Our study also highlights that for students, their respective educational institutes were the main information reservoir for knowledge regarding BFAT, which is consistent with the findings of another study, followed by workshops and the Internet [17]. Keeping that in mind, the Internet should also be used to impart knowledge regarding BFAT and first aid in general to the younger generation, who are more inclined toward using social media platforms rather than traditional sources of information such as television and radio.

While our study has brought into light various significant findings, there are certain limitations to this study. The sample size used was small and not representative of the population under study for the whole country. We used convenience sampling and our sample was not randomized or matched based on gender and age. In addition, the use of a close-ended question means that there might be an overestimation of the participant’s knowledge. Also, we did not assess the participant’s practical skills in performing BFAT, and their knowledge scores may not necessarily translate into their practical performance. Furthermore, the time elapsed since a participant received formal training was also not taken into account, and not compared with their knowledge scores. 

## Conclusions

In our study, there was an overall inadequacy in the knowledge of BFAT amongst undergraduate students. Medical students had better knowledge scores than their non-medical counterparts, and attending a formal training course had a statistically significant impact on the mean knowledge scores of participants. Hence, future larger-scale studies need to be conducted to investigate the various determinants and factors affecting burn first aid practices in Pakistan, in order to reduce the occurrence of this common and highly preventable injury.

## Appendices

First Aid Management of Burns Questionnaire

Please note, all responses provided will remain completely anonymous and confidential. By filling this form, you agree to be a part of the research "Survey on Knowledge of First Aid Management of Burns Amongst Medical and Non-medical Students in a Karachi, Pakistan."

The information provided will be solely used for research purpose(s) only.

* Required

1. Age (years) *

2. Gender *.

Male Female

3. Are you a medical student? *

Yes No

4. If yes, which year of medical school are you in?

1st 2nd 3rd 4th 5th

5. Which of the following, in your opinion, can cause burns? (you can pick multiple options) *

Check all that apply.

Hot water Steam Chemicals Leather and Rubber Paint Flame Electricity Radiation

6. Most common location where burns occur? *

Bedroom Kitchen Bathroom Work Place Don't know

7. What is the MOST IMPORTANT contributor to the severity of burns? *

Time interval between getting burnt and seeking treatment Length of contact Education level of caregiver Method of care given Don't know

8. What is the BEST initial step taken immediately after a burn injury? *

Removing any clothing or accessories Seeking medical assistance Wrapping the burnt area with a clean bandage Cooling burnt area under running tap water Immersing the burnt area in a container of water Applying Toothpaste/honey Applying Ice Don't know

9. What is the optimum time for cooling of the burnt area? *

5 minutes 10 minutes 20 minutes 30 minutes Don't know

10. If a person is wearing any jewelry/ornaments around the burnt area, what should be done? *

Remove it first and then put the burnt area under water Cool under water first and then remove it Cool under a fan Leave it as it is Don't know

11. If a blister forms on the burnt area, what should you do? *

Puncture them as they form Apply toothpaste/Honey Leave them until you seek medical assistance Wrap the burnt area TIGHTLY with a bandage Don't know

12. If the person has burns on the face or neck region, what should be done? *

Immerse the burnt area in a container of water Make the person sit up and reassure them Make the person lie down and reassure them Apply ice over burnt area Don't know

13. How should pain be managed in case of a minor burn injury? *

Put Ice over the burnt area Cold Compresses Over the counter pain medication (analgesics) Warm compresses Don't know

14. If you see a person on fire, what should you do? *

Cover them with a blanket Throw water on the person Call for medical assistance Ask them to stop, drop, and roll Don't know

15. Have you received any formal training/attended any workshop regarding burns and its management? *

Yes No

16. On a scale of 1 to 5, how confident are you in providing First aid to a burn victim? *

1- Not confident at all 2- Slightly confident 3- Moderately confident 4- Confident 5- Very confident

17. From where did you receive knowledge regarding first aid management of burns? (you can

select multiple options) *

Check all that apply.

I do not have any knowledge in this regard Internet Television My educational Institute Workshop/seminar Peers/colleagues/family members Books/journals
